# The Alteration of Chloride Homeostasis/GABAergic Signaling in Brain Disorders: Could Oxidative Stress Play a Role?

**DOI:** 10.3390/antiox10081316

**Published:** 2021-08-21

**Authors:** Provvidenza M. Abruzzo, Cristina Panisi, Marina Marini

**Affiliations:** 1 Department of Experimental, Diagnostic and Specialty Medicine, School of Medicine, University of Bologna, 40138 Bologna, Italy; provvidenza.abruzzo2@unibo.it; 2 Fondazione Istituto Sacra Famiglia, Cesano Boscone, 20090 Milan, Italy; cristina.panisi01@universitadipavia.it

**Keywords:** intracellular chloride concentration, GABAergic signaling, chloride co-transporters, NKCC1, KCC2, neurological and neurodevelopmental diseases, Down’s Syndrome, autism spectrum disorders, bumetanide, oxidative stress

## Abstract

In neuronal precursors and immature neurons, the depolarizing (excitatory) effect of γ-Aminobutyric acid (GABA) signaling is associated with elevated [Cl^−^]_i_; as brain cells mature, a developmental switch occurs, leading to the decrease of [Cl^−^]_i_ and to the hyperpolarizing (inhibitory) effect of GABAergic signaling. [Cl^−^]_i_ is controlled by two chloride co-transporters: NKCC1, which causes Cl^−^ to accumulate into the cells, and KCC2, which extrudes it. The ontogenetic upregulation of the latter determines the above-outlined switch; however, many other factors contribute to the correct [Cl^−^]_i_ in mature neurons. The dysregulation of chloride homeostasis is involved in seizure generation and has been associated with schizophrenia, Down’s Syndrome, Autism Spectrum Disorder, and other neurodevelopmental disorders. Recently, much effort has been put into developing new drugs intended to inhibit NKCC1 activity, while no attention has been paid to the origin of [Cl^−^]_i_ dysregulation. Our study examines the pathophysiology of Cl^−^ homeostasis and focuses on the impact of oxidative stress (OS) and inflammation on the activity of Cl^−^ co-transporters, highlighting the relevance of OS in numerous brain abnormalities and diseases. This hypothesis supports the importance of primary prevention during pregnancy. It also integrates the therapeutic framework addressed to restore normal GABAergic signaling by counteracting the alteration in chloride homeostasis in central nervous system (CNS) cells, aiming at limiting the use of drugs that potentially pose a health risk.

## 1. Intracellular Chloride Concentration Affects the GABAergic Signaling

Intracellular chloride concentration [Cl^−^]_i_ has a critical role in osmotic equilibrium, cell volume regulation [1,2,3,4], acid-base balance [5], and cell signaling [4,6], reviewed in ref. [7,8].

**Figure 1:**
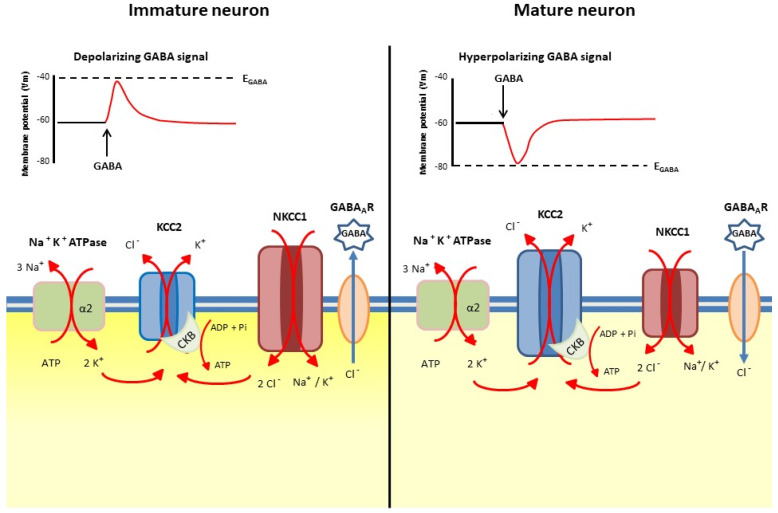
The main cation-coupled chloride co-transporters which mediate [Cl^−^]_i_ developmental shift. Left side: in immature neurons [Cl^−^]_i_ is higher than in mature ones (right side). The intensity of the cytoplasm color refers to the intracellular chloride concentration. KCC2, K^+^-Cl^−^ co-transporter 2; NKCC1, Na^+^, K^+^-2Cl^−^ co-transporter 1; CKB, brain-type creatine kinase; Na^+^, K^+^-ATPase, sodium-potassium pump. The α2 subunit of Na⁺/K⁺-ATPase couples potassium entry into the cell with the K-Cl coordinated efflux from the cell. The size of the symbols depicting KCC2 and NKCC1 is reminiscent of the change of the co-transporter amount and activity in the developmental shift.

Developmentally regulated [Cl^−^]_i_ is the main determinant of the action of GABA at GABA_A_ receptors. In immature neurons, high [Cl^−^]_i_ results in a depolarizing response to GABA, and plays an active role in neuronal growth and the formation of synaptic connections [9,10,11,12]. On the other hand, in mature CNS neurons, a hyperpolarizing or a shunting response is generated by GABA signaling in the presence of low [Cl^−^]_i_ [13]. The K^+^-Cl^−^ co-transporter KCC2 mediates the efflux of Cl^−^ from the cells [14], while the Na^+^, K^+^-2 Cl^−^ co-transporter NKCC1 promotes Cl^−^ uptake [15]. The decrease in [Cl^−^]_i_, which occurs during the maturation of neural cells, is a fundamental ontogenetic process under transcriptional and epigenetic control, which results in a shift of GABAergic transmission from excitation to inhibition [16]. These events occur in an asynchronous way in different brain areas following their sequential maturation, and are correlated with the regulated increase in KCC2 expression, while NKCC1 expression may decrease or remain constant [13,17]. Figure 1 shows the developmental shift in [Cl^−^]_i_, with the main cation-coupled chloride co-transporters involved and the accompanying enzymatic activities needed for their function.

In the brain, KCC2 expression and its regulation are under the control of developmental cues and of brain-derived natriuretic factors (BDNF), through the extracellular signal-regulated kinase1/2(ERK1/2)-dependent upregulation of the transcription factor early growth response 4 (Egr4) [18]. It is worth mentioning that the neurotrophin BDNF plays an important role in cognitive tasks [19]. In a similar way, the insulin-like growth factor-1 (IGF-1) also positively controls KCC2 expression [20] and triiodothyronine, oxytocin, gonadic hormones and other factors affect KCC2 and NKCC1 expression and activity (reviewed in ref. [13]). In particular, the effect of estradiol on KCC2 expression insubstantia nigra pars reticulata neurons differs between males and females [21]. In rat hippocampal and neocortical neurons, oxytocin exerts its effect at birth by post-translational inhibition of NKCC1 [22]. Notably, developmental upregulation of KCC2 begins at different times for various species; for example, occurring during the second half of gestation in humans (reviewed in ref. [23]), and beginning at E18 in rats [24]. The maturation of brain regions sequentially follows the temporal pattern of neurogenesis [24], resulting in a time window for toxicant exposure during ontogenesis [25].

It is worth noting that, besides GABA_A_-R, other chlorine and anion channels contribute to Cl^−^ fluxes across the membrane, the most prominent being glycine receptor (Gly-R) channels, which complement the role of GABA in chloride homeostasis, but may be involved in different processes during brain development [26]. Astrocytes may contribute to the activation of both GABA_A_-R and Gly-R by releasing GABA and the atypical aminosulfonic acid taurine, which activates Gly-R channels [4].

Chloride homeostasis as described here is not universal, as it differs between central and peripheral neurons (reviewed in ref. [27]). In dorsal root ganglion neurons, [Cl^−^]_i_ is higher than in other cell types, since KCC2 expression is barely detectable, whilst chloride efflux is mostly mediated by KCC1 and KCC3, whose activity mostly responds to cell swelling; in addition, [Cl^−^]_i_ is increased by the activity of other anion transporters [27]. Thus, in primary afferent neurons, similarly to immature CNS neurons, opening of Cl^−^ channels usually causes membrane depolarization rather than hyperpolarization. However, the degree and the velocity of depolarization are subject to regulation and determine whether depolarization results into inhibitory or excitatory effects. For instance, intense stimulation of nocireptors and inflammatory mediators can increase [Cl^−^]_i,_ and thus mediate inflammatory hyperalgesia and contribute to neuropathic pain [28]. It is interesting to note that loss-of-function of KCC3 results in Hereditary Sensorimotor Neuropathy with Agenesis of the Corpus Callosum (HSMN/ACC) and axonal swelling [29]. GABAergic neurons are also integrated in the complex cellular network constituting the enteric nervous system (ENS), which regulates, independently of the CNS, gastrointestinal functions, including motility and secretion, and relating with gut microbiota and the enteric immune system. ENS neurons have elevated intracellular Cl^−^ concentration; therefore, activation of GABA_A_ R results in an excitatory effect [30]. Notably, a potential anti-inflammatory role has been suggested for GABAergic signaling in ENS, in synergy with other enteric immunomodulatory mediators [31].

## 2. The Post-Translational Regulation of Cation-Cl^−^ Co-Transporters

The activity of cation-Cl^−^ co-transporters is driven by the Na^+^ and K^+^ gradients generated by the Na^+^, K^+^-ATPase. In particular, Cl^−^ influx is driven by Na^+^, while Cl^−^ efflux is mediated by K^+^ extrusion from the cell. The physical association of KCC2 with the α2 subunit of Na^+^, K^+^-ATPase and with the brain-type creatine kinase (CKB) at the cell membrane couples the generation of ATP by ADP phosphorylation mediated by CKB with the generation of the electrochemical gradient, which drives Cl^−^ out of the cell. In the brain, both CKB and the α2 isoform of the α subunit of the Na^+^, K^+^-ATPase are transcriptionally co-regulated and spatially co-located with KCC2 as reviewed in [13].

The developmental shift in [Cl^−^]_i_ is regulated also by several phosphorylation and dephosphorylation events on both NKCC1 and KCC2. Such post-translational modifications are differentially modulated during development and, in particular, involve the kinases PKC (α, β and γ), WNK (1–4), and SPAK/OSR1, which are downstream of WNKs reviewed in ref. [13]. It is noteworthy that the SPAK/OSR1 pathway is activated by several signals, including estradiol and osmotic stress (i.e., hypertonic conditions), and results in the phosphorylation of both NKCC1 and KCC2; the phosphorylation of the former leads to the enhancement of its activity, whereas the phosphorylation of the latter has a repressive role [32,33]. Such activity likely leads in neurons to an increase in [Cl^−^]_i_ and to the modulation of GABA_A_-R-mediated synaptic inhibition. The role played by WNK and SPAK/OSR1 led Khale et al. [3] to suggest WNK-SPAK inhibition as a potential therapeutic target in neurological diseases not associated with cell swelling.

Chloride co-transporters may also be regulated by their internalization. PKC, by phosphorylating Ser940 of KCC2, contrasts its internalization, thus playing a positive role in KCC2 activity; in turn, PKC is activated by both the metabotropic Glutamate receptor 1 s and by the 5-hydroxytriptamine (5-HT) 2A receptor [34]. These complex interactions are mentioned here to highlight the fact that multiple neurotransmitter receptors are involved in the regulation of neuronal [Cl^−^]_i_; when in the proximity of GABA_A_ receptors, they contribute to the modulation of the GABAergic response, which clearly appears as a key point in both physiology and pathology.

Finally, proteolytic events may modulate the chloride co-transporter amount. It was reported that peripheral nerve injury increases spinal N-methyl-d-aspartate receptor (NMDAR) activity; the consequent increase in [Ca^2+^]_i_ impairs synaptic inhibition through calpain-mediated KCC2 proteolysis [35].

## 3. Intracellular Chloride Concentration and GABAergic Signaling in Neurological Disorders and Neurodevelopmental Diseases and the Involvement of Oxidative Stress and Inflammation

As outlined above, in CNS, both NKCC1 and KCC2 undergo a developmentally regulated alteration in their expression levels in the course of ontogenesis. However, pathological situations might alter the timing and/or the extent of the transcriptional variations of chloride co-transporters. Since GABAergic signals play a key role in orchestrating the assembly of neuronal circuits in the developing brain [36,37] and are the major inhibitory transmitters in the adult brain, it is not surprising that the dysregulation of GABAergic signaling has been associated with many neurological and neurodevelopmental disorders, such as epilepsy, schizophrenia, Down’s Syndrome (DS) and ASD [38,39]. In particular, the so-called Maternal Immune Activation (MIA), by providing a pro-inflammatory intrauterine environment, delays the developmental shift of GABA_A_ receptors [40]. This effect, likely mediated by the pro-inflammatory cytokine IL-1β, which downregulates the transcription of KCC2, is particularly relevant because MIA is one of the more promising murine models of Autistic Spectrum Disorders (ASD) [41], but has also been linked to enhanced risk of schizophrenia in humans [42]. Besides inflammation, other environmental factors may delay the perinatal chloride shift. An example is the effect of bisphenol A on cortical neurons: bisphenol A is an endocrine disruptor, which alters chromatin, negatively affecting the expression of the Kcc2 gene, either directly or following increased MECP2 and decreased H3K9ac binding to Kcc2 regulatory regions [43]. This clearly demonstrates the impact of environmental toxicants on epigenetic machinery in neurodevelopment.

Dysregulation of NKCC1/KCC2 activity ratio is generally the key for improper [Cl^−^]_i_ and alteration of GABA-induced inhibition. Epilepsy, schizophrenia and the neurodevelopmental disorders mentioned above widely differ in their etiology, severity, clinical presentation, age of onset, genetic background, and other features; therefore, it appears somehow inconsequent that this diverse set of conditions could be due to the same pathogenic mechanism: the perturbation of the inhibitory effect of GABAergic signaling.

In the next few paragraphs we will shortly review the evidence, showing that these conditions share two common features: OS and (neuro)-inflammation. In analogy with the well-known phenomenon of glutamate-induced excitotoxicity, it is reasonable to hypothesize that OS and (neuro)-inflammation may be a consequence of the intrinsic alteration of the GABAergic signaling occurring in these neurological and neurodevelopmental disorders. However, instead, we intend here to advance and support the opposite hypothesis, i.e., that OS is the cause rather than the effect of the attenuation or even the reversal of the inhibitory effect of GABA signals at GABA_A_ receptors. We will point out that there are several mechanisms by which OS is able to reduce the expression and the activity of KCC2, thus contributing to the increase in [Cl^−^]_i_ and to the global excitability of CNS cells.

The classical definition of OS was formulated by Halliwell and Guteridge in 1989 [44]: “Oxidative stress occurs due to the imbalance between the production of Reactive Oxygen Species (ROS) and the availability of antioxidants or radical scavengers”. However, by taking into account that ROS play a significant role not only as potential damaging agents but also as signaling molecules, a more comprehensive definition was put forward by Jones in 2006 [45]: “Oxidative stress may be better defined as a disruption of redox signaling and control”. ROS production occurs at many cellular sites and in manifold and varied conditions, both physiological and pathological. In particular, dysfunctional mitochondria are a relevant source of ROS [46].

In turn, inflammation is often a result of OS [47]. In particular, Advanced Glycosylation End-products (AGE)-derived from oxidized proteins, nucleic acids and lipids, may be a direct cause of inflammation; moreover, both ROS and AGE may activate inflammatory signaling cascades, sometimes leading to cell death, autophagy and inflammasome activation [48,49,50].

As pointed out by Kim et al. [51], the evidence suggests that major neuropsychiatric disorders, such as autism spectrum disorder (ASD), schizophrenia, bipolar disorder and major depressive disorder, share a number of pathogenic mechanisms based on the interaction between the environment and the genome, leading to the impairment of brain energy metabolism, mitochondrial functions and redox balance. In support of these claims, they report that alterations of the mitochondrial genome in psychiatric patients have been described [52,53], as well as several mutations and polymorphisms of mtDNA, decreased oxidative phosphorylation and increased generation of hydrogen peroxide in lymphocytes from ASD children [54]. Moreover, they point out that mitochondrial dysfunctions, in addition to generating ROS, affect mitochondrial dynamics, circadian rhythms and glutamatergic transmission. Other mechanisms of mitochondrial dysfunction in ASD are linked to dysbiosis and to environmental toxicants [52].

### 3.1. Epilepsy

Epilepsy is a devasting neurological disorder, affecting more than 70 million people worldwide [55]. It is characterized by recurrent seizures, due to excessive and abnormal neuronal activity in the brain cortex. About one third of epilepsy patients suffer from uncontrolled seizures despite pharmacotherapy [56]; therefore, a better insight into the pathological mechanisms is needed. Epilepsy may originate from various conditions, ranging from genetic and birth defects to brain injuries and tumors.

The importance of excitatory–inhibitory imbalance in epileptogenesis has been reviewed by Liu et al. [57], who reported the observation of upregulation of NKCC1 and/or dowregulation of KCC2 (mRNA and protein) in different animal models of chronic epilepsy. In a similar way, the epigenetic dysregulation of chloride co-transporters has been reported to occur in juvenile myoclonic epilepsy [58]. Epileptogenic brain tumors also exhibit a shift in GABAergic signaling from inhibitory to excitatory, as reviewed in ref. [57]. In support of the hypothesis of the role of the cation chloride co-transporters in the generation of seizures, a number of researches reported the attenuation of epileptic activity operated in humans [59,60] and in several animal models [57] by bumetanide, an inhibitor of NKCC. In an interesting work on human epilepsy, Kipnis et al. [61], while identifying the origin of seizure susceptibility in alterations of the chloride co-transporters, stressed the relationship between their developmental regulation operated by BDNF and gonadic hormones with sexual dimorphism observed in epileptogenesis. In addition, it has been reported that alteration of signals delivered at the synaptic metabotropic zinc receptor mZnR/GPR39, which physiologically enhance KCC2 activity, is associated with epileptic seizures [62].

Among the possible causes of chloride co-transporter unbalance, rare loss-of-function mutations of the SLC12A5 gene, encoding KCC2, were reported in patients with epilepsy [63]. Altered epigenetic regulation of genes encoding chloride co-transporters seems more plausible than rare genetic mutations in determining an improper [Cl^-^]_i_ unbalance in some brain areas. The methylation state of NKCC1 and KCC2 genes has been assessed in juvenile myoclonic epilepsy patients, showing significantly lower NKCC1 DNA methylation and significantly higher KCC2 DNA methylation levels [58].

Inflammation is one of the most important mechanisms in epileptogenesis and is sustained by the activation of NF-κB (nuclear factor kappa-light-chain-enhancer of activated B cells) signaling and by OS, which justifies the proposal of anti-inflammatory and antioxidant drugs for its treatment [64].

Evidence supporting a role for ROS and for mitochondrial impairment in epilepsy has been reviewed in two recent independent papers [65,66]; in particular, Kovac et al. [65] highlighted the involvement of nicotinamide adenine dinucleotide phosphate (NADPH) oxidases in both ROS generation and mitochondrial damage, which links ROS generation to microglia activation in a variety of brain diseases.

### 3.2. Schizophrenia

According to the definition of the American Psychiatric Organization, schizophrenia is a severe neurological disease, characterized by delusions, hallucinations, disorganized speech, trouble with thinking and lack of motivation (https://www.psychiatry.org/; accessed on 30 May 2021). Schizophrenia affects males 1.4 times more frequently than females [67].

Rare genetic mutations of cation-coupled chloride co-transporters or of elements of the WNK-SPAK/OSR1 Kinase Signaling Pathway have been identified in several neuropsychiatric and neurodevelopmental diseases reviewed in ref. [68]. Loss-of-function mutations of the SLC12A5 gene, encoding KCC2, and a gain-of-function missense variant in SLC12A2, encoding NKCC1, were described by Merner et al. [69,70]; both variants were linked to schizophrenia and the former also to ASD. Moreover, autoptic samples from schizophrenic patients showed higher transcript levels of Wnk3 and of Oxsr1, encoding OSR1 kinase [71]. Again, a pharmacological intervention aimed at restoring the correct balance of chloride co-transporters, which confirmed the involvement of this mechanism in schizophrenia and in ASD. The administration of bumetanide relieved some symptoms in schizophrenic [72] and ASD patients ([73,74,75], reviewed in ref. [76].

A Special Issue of Schizophrenia Research was devoted in 2016 to OS and inflammation in schizophrenia. In their editorial, Sawa and Sevlak [77] outlined the evidence reported by the seven manuscripts included in the issue, which cover topics such as the detection of OS and inflammatory makers in sera from schizophrenic patients and the influence of redox dysregulation and inflammation on neurotransmission [78]. Further studies were reported in a review by Koga et al. [79]. More recently, two manuscripts highlighted the interplay between OS and cellular markers of inflammation in schizophrenic patients [80,81].

### 3.3. Down’s Syndrome

Down’s Syndrome (DS) is a genetic disorder caused by chromosome 21 partial or full trisomy. It is to all respects a neurodevelopmental syndrome, causing, among other defects, morphogenetic brain anomalies, which in turn lead to intellectual disability and learning difficulties. At the same time, since most DS patients experience in adult life a progressive cognitive decline with neuropathological features consistent with the Alzheimer’s disease phenotype [82,83], DS is often considered a neurodegenerative disorder.

As in other brain disorders, GABAergic signaling was reportedly altered also in DS, with a reduction in GABA concentrations and an increased expression of NKCC1 in the brain of fetuses and children with DS and in the Ts65Dn mouse model of chr. 21 trisomy [84,85,86].

OS was first described in 1989 to occur in DS cells [87]. It was then recognized to be caused by mitochondrial dysfunction, which by itself has profound consequences on ATP production and deficit in total brain energy reviewed in ref. [88]. In turn, mitochondrial-originated ROS caused a progressive accumulation of oxidative damage in mtDNA, leading to early cellular aging and neurodegeneration, as reviewed in ref. [68]. Low levels of BDNF were described in the hippocampi of human fetal brains from individuals with trisomy 21 [89]. Interestingly, by utilizing a murine transgenic model of DS carrying an extra copy of DYRK1A (a candidate gene for DS), which displays both brain abnormalities and learning impairment, Guedj et al. [89] demonstrated that the overexpression of DYRK1A was responsible for the decrease in BDNF: transgenic mice fed with the DYRK1A inhibitor epigallocatechin gallate (EGCG)—a member of a natural polyphenols family from green tea—increased BDNF expression and partially rescued cognitive deficits. Many flavonoids have well-established antioxidant and free-radical-scavenging activities, but also exert their biological effects through direct actions on enzymes, receptors and signaling pathways; they are also endowed with a modulatory effect on GABA_A_ receptors [90]. Neurodevelopmental abnormalities in DS include several impairments in neurotransmission systems; not only is the GABAergic signaling altered, but also the glutamatergic, the cholinergic and the serotonergic pathways are reviewed in ref. [85].

### 3.4. Autism Spectrum Disorder

Autism Spectrum Disorder (ASD) is a neurodevelopmental disorder characterized by impaired social communication skills, restricted interests and activities, repetitive patterns of behavior and sensory abnormalities, including anomalous pain sensitivity [91,92]. Intellectual disability is reported in half of the people with an ASD diagnosis [93]. About 5–46% of individuals with ASD also suffer from epilepsy [94]. The prevalence of autism is strongly unbalanced with regard to gender, being diagnosed in about four males for each female; the reason for such sexual dimorphism is still a matter of debate [95]. Autistic features are found also in people with genetic syndromes affecting neurodevelopment, including Rett and Fragile X syndromes; therefore, for the purpose of this review, we will deal with these conditions together with ASD. The genetic architecture is complex in ASD and the lack of demonstration of causal genetic abnormalities leads to defining the majority of cases as “idiopathic” [96]. The wide variability of behavioral manifestations and the frequent multisystem involvement—disorders in ASD people are not limited to the nervous system—suggest that epigenetic mechanisms impact on the neurodevelopment during the ontogenesis and in the first two years of life, which is the period of maximum neuroplasticity as reviewed in ref. [97]. Furthermore, an epigenetic model also accounts for the steep worldwide increase in ASD prevalence in the last decades, within the framework of the epidemiological transition towards a greater predominance of non-communicable diseases [98,99]. It is also of note that idiopathic forms of ASD often share disregulated gene expression/pathways with syndromic (monogenic) forms; this strongly suggests that, in idiopathic ASD forms, alterations of chromatin remodeling may mimic genetic mutations [100].

As mentioned above, in mature neurons, Gly-R contributes to the establishment of [Cl^-^]_i_, which results in the hyperpolarizing action of GABA_A_ receptors. It is interesting to note that a number of rare mutations affecting Gly-Rs are associated with ASD [101]. At inhibitory synapses, glycine and GABA_A_ receptors are anchored to the cytoskeleton by the scaffold protein gephyrin, which contributes to the post-synaptic clustering of GABA_A_ receptors. Several gephyrin alterations reviewed in ref. [102], are associated with neuropsychiatric and neurodegenerative disorders, including ASD and schizophrenia. Gephyrin with the G375D missense mutation has a reduced binding affinity to GABAA and GlyRs and was found in a patients with epileptic encephalopathy [103].

Carriers of some genetic variants of the Cystic Fibrosis Transmembrane Conductance Regulator (CFTR) may present with cystic fibrosis associated with ASD, probably because CFTR may be a positive regulator of NKCC1 [104]. ASD is not the only neurodevelopmental disorder where GABAergic signaling appears to be compromised. In fact, GABAergic signaling is reportedly altered also in patients carrying either of two genetic mutations that share many clinical features with ASD, namely Rett syndrome [105,106] and Fragile X syndrome [107].

Recalling the modulatory role of numerous and diverse neurotransmitter receptors localized at the GABAergic synapses, some examples where their alteration affects neurodevelopmental disorders are worth mentioning. In fact, 5-HT receptors are affected by the absence of the Fragile X mental retardation protein [108] and the abnormal methylation of 5-HT transporter gene network is associated with Attention Deficit Hyperactivity Disorder (ADHD) and with early life stress [109]. In addition, agonists of the metabotropic acethylcholine receptor reduce stereotypies in BTBR mice (a model of ASD) [110].

The first evidence that ASD was associated with OS dates back to 2004–2006 [111,112,113] and were corroborated by further studies showing that OS altered the lipid asset of erythrocyte membranes [114,115]. In addition, OS altered also the erythrocyte shape, both in ASD and in Rett patients [116,117]. Metabolomic analysis of ASD plasma and urine revealed the presence of elevated levels of AGE [118]. Several studies link OS to mitochondrial dysfunctions [119], to immune dysregulation and inflammation [120] and to gut dysbiosis, often accompanied by severe gastrointestinal problems [121,122], as reviewed in ref. [97]. Lower expression of BDNF was found in the neonatal blood of children, which were diagnosed with ASD later in life [122,123]; data from a murine model of ASD suggest that BDNF levels may be epigenetically regulated in a sexually dimorphic fashion [124]. It is worth noting that a complex relationship links BDNF expression and MeCP2, the gene whose mutation is the cause of Rett syndrome [125]. Another neurotrophic factor, IGF-1, which activates the PI3K/AKT pathway, is dysregulated in both idiopathic and syndromic ASD [19,126]. In particular, in the age range 1–4 years, the amount of BDNF in cerebrospinal fluid was significantly lower than in neurotypical children [127]; intense research is currently underway to evaluate a therapeutic use of IGF-1 in ASD [128,129].

## 4. How OS, Inflammation, Toxicants and Chromatin Modifiers May Affect Intracellular Chloride Concentration and GABAergic Signaling

As mentioned above, OS ensues when ROS production overwhelms the antioxidant capacities of cells or tissues; in contrast, in physiological conditions, ROS play a significant role as signaling molecules. The presence of cysteine residues in some GABA_A_ receptor subunits, both on the extracellular and the intracellular loops, potentially makes GABA_A_ receptors susceptible to ROS-operated modulation of ion channel gating.

Exposure of GABA_A_ receptors to artificially elevated levels of ROS, either extracellular or intracellular, did not seem to affect the intracellular chloride concentration, since, at least in the hippocampus and in the cerebellum, ROS potentiated the inhibitory activity of GABA_A_ receptors as reviewed in ref. [130]. However, it is worth considering that we ignore whether such experiments gave an insight into the physiological role of ROS signaling or rather simulated OS by unbalancing the redox state of the cells. Also inconclusive in this regard was the work of Toczylowska et al. [131], who studied hippocampal metabolite profiles by proton nuclear magnetic resonance spectroscopy, comparing two murine models of pharmacologically induced ASD: valproate and thalidomide. Globally, the changes in metabolites suggested disturbances in excitatory and/or inhibitory neurotransmission, in energy production and in membrane lipid composition, along with the presence of OS. While OS might justify the decrease in energy production and changes in lipid composition, the study could not discriminate whether excitatory–inhibitory unbalance was a cause or a consequence of OS.

Other evidence supports a role for OS in the alteration of the excitatory/inhibitory balance at GABA_A_ receptors in pathological models. Hepatic encephalopathy is an example of a medical condition that may indirectly alter the redox equilibrium of CNS. It causes a motor dysfunction, originating in the substantia nigra pars reticulata (SNr), a midbrain region where the GABAergic neurons convey the final processed signals of the basal ganglia to the thalamus and superior colliculus. Bai et al. [132] reported that OS made the GABA signals lose their inhibitory effect in SNr and that such a dysfunction was accompanied by a decreased expression of the chloride co-transporter KCC2. By inhibiting the oxidative processes and reducing ROS contents, Bai et al. improved the dyskinesis caused by OS and restored normal levels of KCC2.

As discussed above [40], KCC2 expression is reduced also as a result of inflammatory stimuli, via chromatin modifications induced by pro-inflammatory cytokines. It was observed that increased levels of maternal cytokines and chemokines during gestation are associated with the development of autism in the offspring [133]. Moreover, a pro-inflammatory signature persisting after birth and can be found in ASD children [134]. Besides maternal immune stimulation, other stressful events, such as physical constraint or maternal separation of puppies, causes the increase of pro-inflammatory cytokines, as recently reviewed by Pozzi et al. [135].

Similar results are observed following exposure to toxicants. The above-mentioned bisphenol A, an endocrine disruptor [43], has been causally associated with the development of ASD [136]. In turn, perinatal exposure to Pb^2+^ subverts the KCC2/NKCC1 ratio, albeit through mechanisms not completely known [137]; moreover, it is worth mentioning that Pb^2+^ induces both oxidative stress and inflammation and causes a decrease in cerebellar BDNF [138]. Exposure of fish brain to Pb^2+^ demonstrated increased production of reactive oxygen species, increased lipid peroxidation, loss of protein thiol groups in synaptosomal fraction, decreased activity of Na^+^, K^+^-ATPase, partial inactivation of mitochondrial electron transport chain activity and energy depletion [139]. Scientific literature abounds with evidence that exposure to heavy metals, toxicants and endocrine disruptors may cause oxidative stress and inflammation and is associated with neurodegenerative and neurodevelopmental disorders [120,140].

Two further aspects relate the perturbation of [Cl^-^]_i_ to oxidative stress and are grounds for reflection when considering the impact of GABAergic signaling in neurodevelopmental disorders. One is the above-mentioned activation of OSR1, which, by phosphorylating both NKCC1 and KCC2, enhances and represses their activities, respectively. OSR1 stands for “oxidative-stress responsive-1” and, in humans, it is encoded by the gene OXSR1. Since it is 39% identical to human SOK1 (Ste20/oxidant stress response kinase-1), a molecule that is activated by oxidative stress (OS) [141], it was hypothesized that OSR1 was also activated by OS, in the absence of any direct proof about an OS-dependent activation of OSR1. However, more recently, a global quantitative phosphoproteomics approach to identify cytoplasmic proteins altered in their phosphorylation state by an ataxia-telangiectasia-mutated kinase (ATM) detected OSR1 as a novel substrate of ATM after hydrogen peroxide exposure of cells [142]. OSR1-driven post-translational modifications of chloride co-transporters have long been seen in the context of cellular osmotic control; however, the observation that OSR1 may be activated also by oxidative stress [142] opens new perspectives to the post-translational control of chloride co-transporters. Accordingly, recent work [143] has demonstrated that two different animal models of autism—a model of Rett syndrome and the prenatal exposure to valproic acid—showed increased levels of ATM and decreased egr4 activity on the Kcc2b promoter, leading to decreased expression of Mecp2 and a delayed GABAergic developmental shift. Treatment with KU55933, an ATM inhibitor, was able to restore normal levels of KCC2, rescuing abnormal GABAergic signaling and autism-like behavior in mice. This is another example of how the autistic phenotype may be a consequence of different triggers, converging to the alteration of the same pathways. The presence of an oxidative environment may mimic specific genetic or chemical triggers by favoring decreased expression/activity of KCC2, with the consequent alteration of GABAergic signaling.

The second aspect relies on previous work on the relevant decrease of Na^+^, K+-ATPase activity in both erythrocytes and leukocytes of ASD children [114,144], which pointed out the importance of the membrane context embedding the Na^+^, K^+^-ATPase. Reduced membrane cholesterol, and oxidative stress-induced damage to membrane lipids, play crucial roles in decreasing the Na^+^, K+-ATPase activity also in schizophrenia [145]. Note that at variance with blood cells, brain Na^+^, K^+^-ATPase expresses the α2 subunit, which functionally interacts with KCC2 [146], and was reported to mediate nuclear factor kappa B (NFκB) signaling in LPS-induced immune response [147]. In addition, it should be stressed that OS often results from dysfunctional mitochondria and, in turn, impairs mitochondrial functioning, in a vicious loop reviewed in ref. [97]. As a result, cellular energy supplies are jeopardized, whilst they are required to fuel chloride efflux at the KCC2 co-transporter, which depends on the continuous supply of ATP, mediated by the membrane-embedded brain-type creatine kinase CKB.

**Figure 2:**
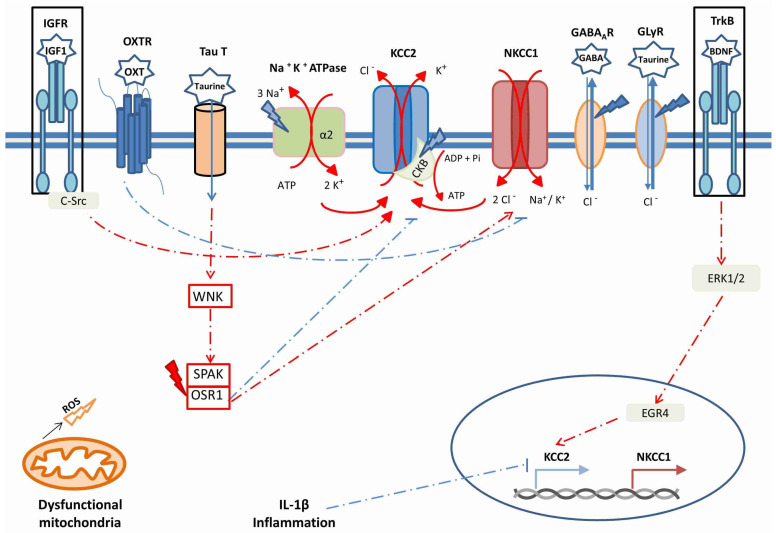
The main factors modulating KCC2 and NKCC1 synthesis and activity, in relation with anomalies described in neurological and neurodevelopmental disorders. Oxidative stress/ROS is symbolized by lightning bolts; blue bolts indicate a loss-of-function effect, red bolts indicate a gain-of-function effect. The boxes surrounding IGFR and TrkB indicate that IGF-1 and BDNF signaling are both decreased in ASD and in other neurodevelopmental disorders.

The relationships between OS, inflammation and maintenance of [Cl^−^]i are shown in Figure 2.

## 5. Conclusions (and Therapeutic Suggestions)

As pointed out by several Authors, [Cl^−^]_i_ is instrumental in the effect of GABA at GABA_A_ brain receptors, which undergo a developmental shift from excitatory to inhibitory in a time-and-area-dependent fashion. Disruption of intracellular chloride homeostasis within CNS has profound consequences on the etiopathogenesis of several widespread neurological and neurodevelopmental diseases. Differential control of CNS [Cl^−^]_i_ in males and females during development may provide an explanation of the gender bias in neurobiological disorders, such as ASD and schizophrenia. We outlined here the main evidence linking [Cl^−^]_i_ maintenance with the occurrence of OS and inflammation and, in some way, with exposure to toxicants and chromatin modifiers, in patients with neurological and neurodevelopmental diseases. In addition, several studies even suggest that OS and inflammation might be causative in the disruption of the mechanisms maintaining the correct intracellular chloride concentration. Even if the alteration of GABAergic signals cannot be considered as the only determining aspect in the etiopathogenesis of neurodevelopmental diseases, it is indubitable that the link between GABAergic signals and brain malfunction is important. In fact, this is the rationale for pharmacological modulation of GABA function in ASD [148] and for efforts to pharmacologically control the chloride transporters in the CNS of subjects with DS and ASD, as previously mentioned for bumetanide [73,74,75,76]. Since bumetanide is a diuretic drug, prescribed to reduce symptoms of fluid retention or edema in people with congestive heart failure, liver or kidney disease, as such, is not devoid of side effects, as it inhibits both the ubiquitous isoform NKCC1 and the kidney-specific NKCC2, with consequent diuretic effects. This problem may be overcome by a newly discovered molecule, which selectively inhibits NKCC1, sparing the kidney-specific NKCC2 [149]. In any case, pharmacological targeting of chloride cotransporters might be beneficial for CNS but have negative effects for peripheral neurons, including ENS, where the activity of NKCC1 prevails. In ASD persons, pain sensitivity perturbances and gastroenteric inflammatory symptoms are quite common, which might point to altered GABAergic signaling also in the periphery. This suggests greater caution in the use of drugs targeting the GABA system.

The striking increase in neurological disorders occurring in the last decades represents a relevant issue for the worldwide healthcare system. In particular, major worries relate to the rise of neurodevelopmental disorders, which are usually diagnosed too late to allow intervention within two years of life and is the crucial time window for neuroplasticity [97]. The logical and experimental links shown in the present review between [Cl^−^]_i_ dysfunction and occurrence of OS and inflammation highlight a novel mechanism capable of accounting for the impact of OS and mitochondrial dysfunction on neurodevelopment and the role of OS in the pathophysiology of numerous neurological diseases. As a consequence, these reflections strengthen the suggestion to place most effort into controlling OS and immune activation in pregnant mothers, infants and toddlers. In addition, since [Cl^−^]_i_ dysfunction, and consequential OS and inflammation, are sometimes the result of exposure to toxicants and chromatin modifiers, a large space opens up for prevention [97,140].

The fact that dysfunctions in GABAergic signaling are a common pathogenetic trait shared by a large array of brain disorders highlights the presence of psychopathological and neurological co-occurrences in many neurodevelopmental disorders. Transition of persons with ASD to adulthood is often accompanied by high rates of psychiatric comorbidities [150]. In prefrontal cortical regions, parvalbumin-expressing GABAergic interneurons normally mature during adolescence, and are particularly affected during their development by oxidative stress, neuroinflammation and NMDAR hypofunction [78]; in fact, in patients with schizophrenia, autism and bipolar disorders, they exhibit a pattern of gene expression typical of immature cells [151]. The physiological maturation trajectory of these interneurons suggests that a transition to adult life may pose a demanding challenge to patients where OS is present.

As far as treatment is concerned, in neurological disorders in which the perturbation of GABAergic signaling is supposed, antioxidant molecules and the support of mitochondrial function seem to be the first logical line of intervention. In a different context, the combination of idebenone and tocotrienols gave promising results [152]. Furthermore, even in the case of the prescription of specific drugs targeted to brain chloride co-transporter NKCC1-i.e., bumetanide-, an integrated approach including antioxidant intervention should be considered.

In conclusion, our study proposes the modulation of GABAergic signaling by OS as a paradigmatic situation in which suggestions provided by pathophysiology can inform the clinical perspective. In an era of personalized medicine, the biological complexity of humans makes it unlikely that tailored answers can be represented by single interventions, but rather by the capability to embrace complexity and integrate interventions.
